# Adverse Perinatal Outcomes of True Knot of the Umbilical Cord: A Case Series and Review of Literature

**DOI:** 10.7759/cureus.26992

**Published:** 2022-07-18

**Authors:** Ishita Agarwal, Sweta Singh

**Affiliations:** 1 Obstetrics and Gynecology, All India Institute of Medical Sciences, Bhubaneswar, Bhubaneswar, IND

**Keywords:** perinatal outcome, perinatal mortality, adverse perinatal outcomes, human umbilical cord, true knot

## Abstract

A true knot of the umbilical cord (TKUC) is an actual knot formed in pregnancy. It is seen in approximately 0.3%-1.2% of all pregnancies. True knots are of significance as they can cause a wide spectrum of adverse perinatal outcomes like small for gestational age (SGA) fetus, low appearance, pulse, grimace, activity, and respiration (Apgar) score at birth, fetal hypoxia, and even fetal demise.

Here, we report a case series of three patients with TKUC and the varied adverse perinatal outcomes associated with them. A low-risk primigravida at term gestation had a suspicious non-stress test (NST). Repeat NST after maternal resuscitation became pathological. Emergency cesarean delivery was performed in view of pathological NST persisting despite intrauterine resuscitation. A healthy male baby weighing 2920 g was delivered, and the umbilical cord had a true knot.

A multigravida at 33 + 3 weeks of gestation was referred with fetal growth restriction (FGR). Color Doppler examination showed absent end-diastolic flow (AEDF) in the umbilical artery (UA). Cesarean delivery was performed in view of FGR stage two with AEDF in the UA at 34 weeks of gestation as per the Barcelona criteria. A male baby weighing 1505 g was delivered. The umbilical cord had a true tight knot. The baby had an Apgar score of 7 at one minute after birth but was shifted to the neonatal intensive care unit (NICU) in view of low birth weight and prematurity. The baby slowly gained weight and was discharged from NICU after 15 days in stable condition.

A multigravida at 32 weeks of gestation was referred with intrauterine fetal demise. Ultrasonography confirmed the presence of a single intrauterine dead fetus corresponding to 30 + 4 weeks of gestation with an estimated fetal weight (EFW) of 1633 g, amniotic fluid index (AFI) equal to nine, and presence of Spalding’s sign. Induction of labor was done, and she expelled a dead macerated male fetus weighing 1825 g. The infantogram was normal. A true umbilical cord knot was found.

The umbilical cord is the source of fetal blood supply; therefore, any cord abnormality can have a significant impact on the fetal outcome. There are various factors that can predispose to TKUC, such as polyhydramnios, increased cord length, monoamniotic twins, male baby, grand multiparity, small fetus, and amniocentesis. TKUC can lead to various adverse outcomes in pregnancy and labor like SGA fetus, low Apgar score at birth, fetal hypoxia, and fetal demise. TKUC increases the risk of fetal demise by as much as four times.

With the development of advanced techniques such as three-dimensional/four-dimensional color Doppler ultrasounds, TKUC can be diagnosed antenatally in the form of a four-leaf-clover, a “hanging-noose sign,” or by an unusual multicolor pattern in the cord. The prenatal detection rate of TKUC is only 12%. It mostly remains undetected unless visualized incidentally. Although TKUC is not rare and can have serious outcomes, the importance of its antenatal diagnosis has not been determined. It should be suspected in patients with risk factors, and emphasis should be placed on its antenatal diagnosis on ultrasonography to avoid obstetric disasters in otherwise low-risk females. Though there is no specific management of these cases, a good clinical outcome can be achieved if TKUC is diagnosed antenatally and monitored closely until fetal maturity is attained.

## Introduction

A true knot of the umbilical cord (TKUC) is an actual knot formed during pregnancy, while a false knot refers to a bulge in the cord occurring due to exaggerated looping of cord vessels or excessive covering of Wharton’s jelly [1]. TKUC is seen in approximately 0.3%-1.2% of all pregnancies [2]. True umbilical cord knots are of significance as they can lead to a wide spectrum of adverse perinatal outcomes such as small for gestational age (SGA) fetus, low appearance, pulse, grimace, activity, and respiration (Apgar) score at birth, fetal hypoxia, and even fetal demise [3,4].

In this article, we report a case series of three patients with TKUC and the varied adverse perinatal outcomes associated with them.

## Case presentation

Case 1

A 27-year-old low-risk primigravida at 38 + 2 weeks’ gestation presented with abdomen pain of one-day duration, which was not associated with bleeding or leaking per vaginum. Her antenatal period was uneventful. At the time of presentation, she was afebrile, her pulse rate was 79 beats per minute (bpm), and her blood pressure was 120/80 mmHg. On abdominal examination, the uterus was irritable, and fetal heart rate (FHR) was 170 bpm. On per vaginal examination, the cervix was three centimeters in length, firm, and posterior, and the internal os was closed.

**Figure 1:**
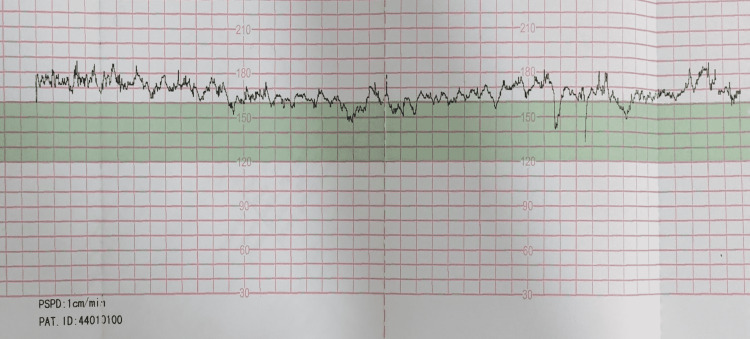
Admission non-stress test (NST) showing baseline fetal tachycardia

Bedside obstetric ultrasound revealed a single live intrauterine fetus in cephalic presentation with an estimated fetal weight (EFW) of 3023 g, FHR of 166 beats per minute (bpm), and amniotic fluid index (AFI) of 14.5 cm. An admission non-stress test (NST) was performed, which showed a baseline FHR of 166-170 bpm, normal beat-to-beat variability, with no accelerations (Figure 1).

**Figure 2:**
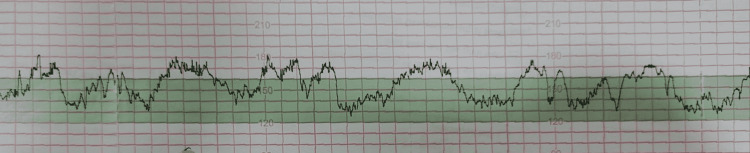
Repeat NST showing persistent fetal tachycardia with multiple spontaneous decelerations

In view of suspicious NST, intrauterine resuscitation was given in the form of hydration, and she was placed in the left lateral position. A repeat NST was performed, which was suggestive of persistent fetal tachycardia of 170 bpm, with variability of 5-25 bpm but with the presence of multiple spontaneous decelerations of ≥15 bpm lasting for ≥15 seconds (Figure 2).

**Figure 3:**
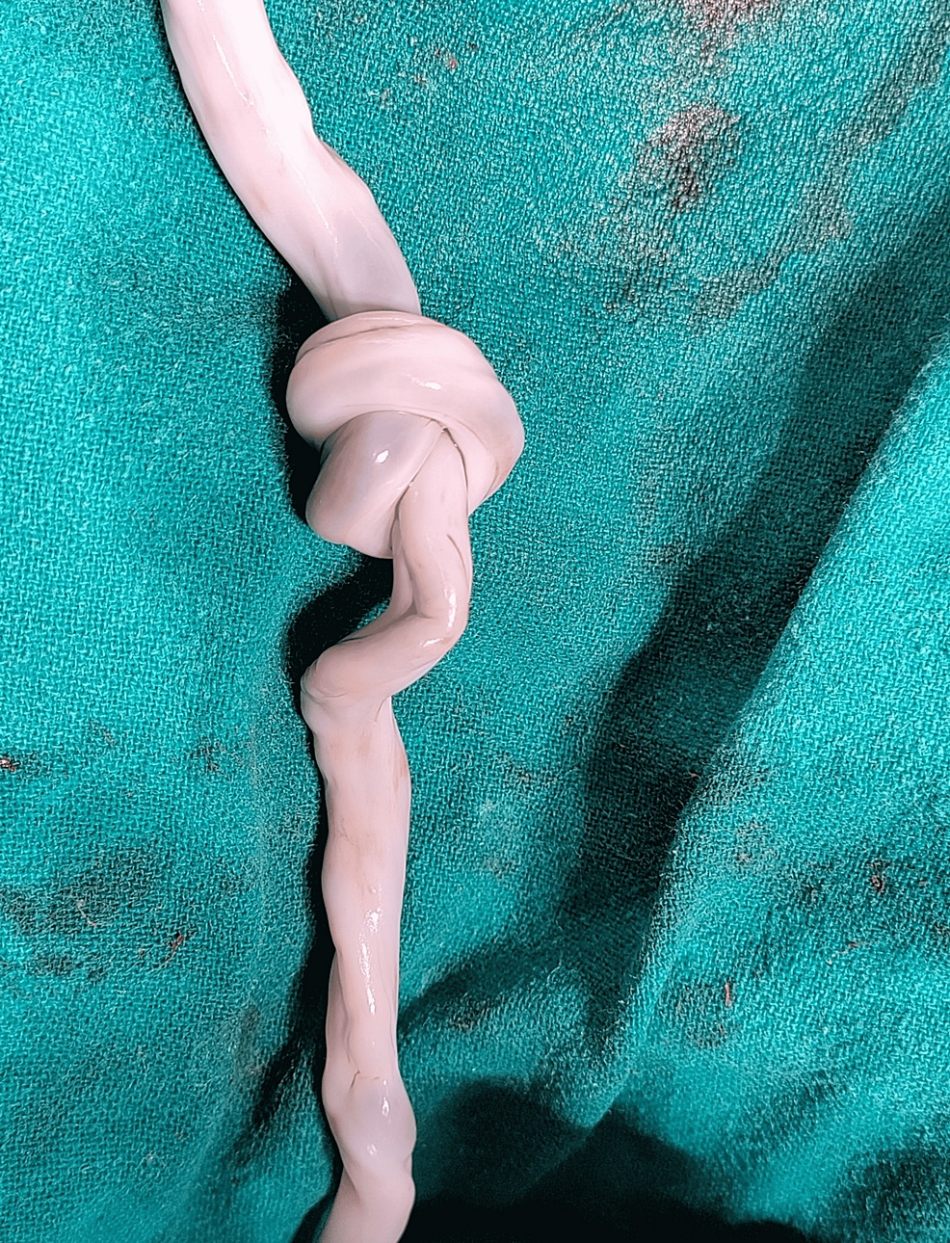
True knot of umbilical cord

Emergency cesarean delivery was performed in view of pathological NST persisting despite intrauterine resuscitation. A male baby of 2920 g was delivered. The baby cried immediately after birth and was handed over to the mother’s side. The umbilical cord had a true knot (Figure 3).

The postpartum period was uneventful, and both the mother and baby were discharged on day four of puerperium in healthy condition.

Case 2

**Figure 4:**
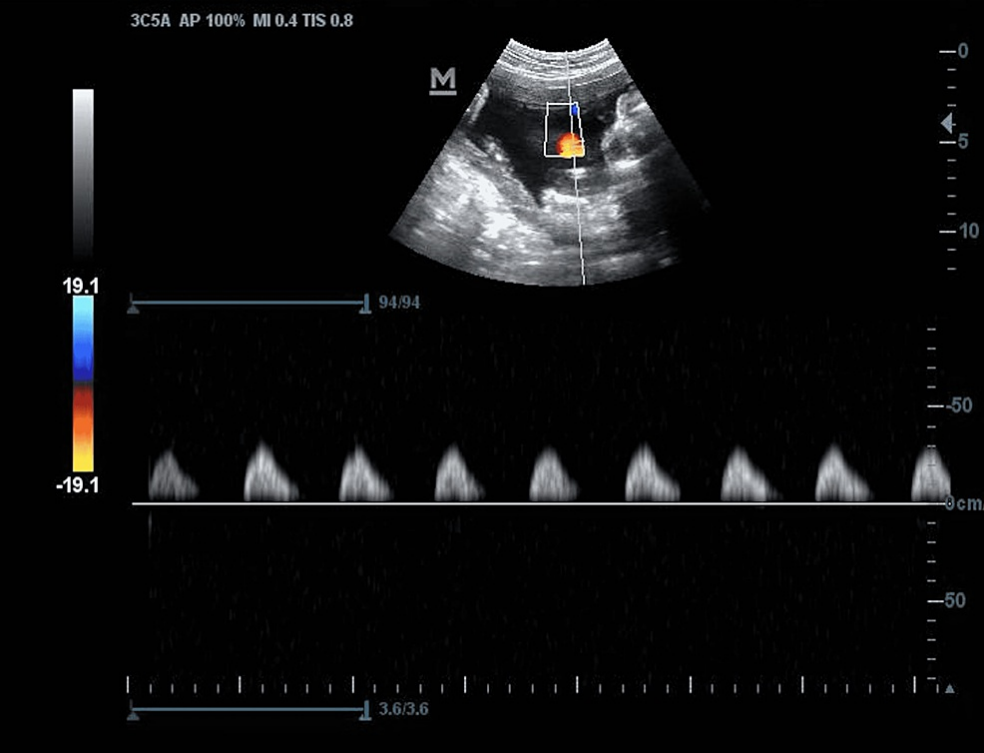
Absent end-diastolic flow in the umbilical artery on Doppler ultrasonography

An unbooked 25-year-old multigravida was referred at 33 + 3 weeks of gestation in view of FGR. Her antenatal period was uneventful. On general examination, her vitals were stable. On per abdomen examination, fundal height corresponded to 28 weeks’ gestation. Fetal biometry by ultrasonography showed EFW of 1428 g, FHR of 137 bpm, and AFI of 10.55 cm. In view of EFW below the third centile, evaluation by Doppler velocimetry was performed, which showed absent end-diastolic flow (AEDF) in the umbilical artery (UA) (Figure 4).

**Figure 5:**
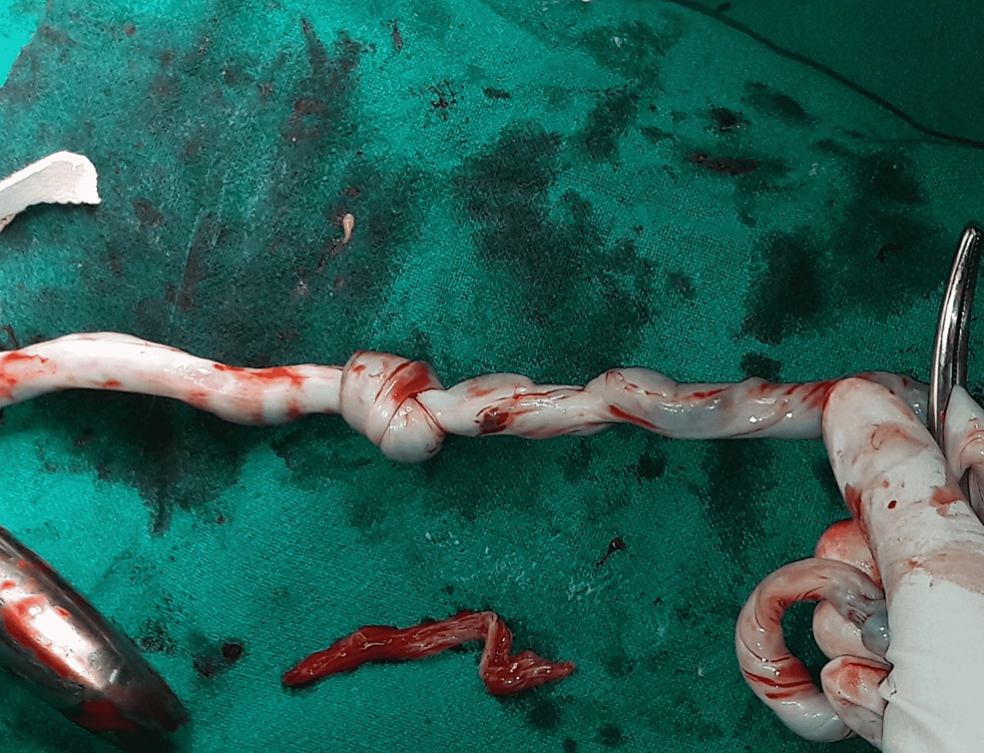
Tight true knot in umbilical cord

She was admitted and investigated. No high-risk factors for the cause of FGR could be identified, and a provisional diagnosis of idiopathic FGR was made. Cesarean delivery was performed in view of FGR stage two with AEDF in UA at 34 weeks of gestation as per the Barcelona criteria. A male baby of 1505 g was delivered, and a true tight knot in the umbilical cord was found (Figure 5).

The baby had an Apgar of seven at one minute after birth but was shifted to the neonatal intensive care unit (NICU) in view of low birth weight (LBW) and prematurity. The baby slowly gained weight and was discharged on day 15 post-delivery in stable condition.

Case 3

An unbooked 32-year-old multigravida with previous cesarean delivery was referred at 32 weeks of gestation with intrauterine fetal demise. Upon presentation, she was afebrile, her pulse rate was 98 bpm, and her blood pressure was 148/94 mmHg. On per abdominal examination, fundal height corresponded to 32 weeks, uterus was relaxed, there was no scar tenderness, and FHR could not be localized. Ultrasonography confirmed a single intrauterine dead fetus corresponding to 30 + 4 weeks of gestation with an EFW of 1633 g, AFI of nine, and presence of Spalding’s sign.

**Figure 6:**
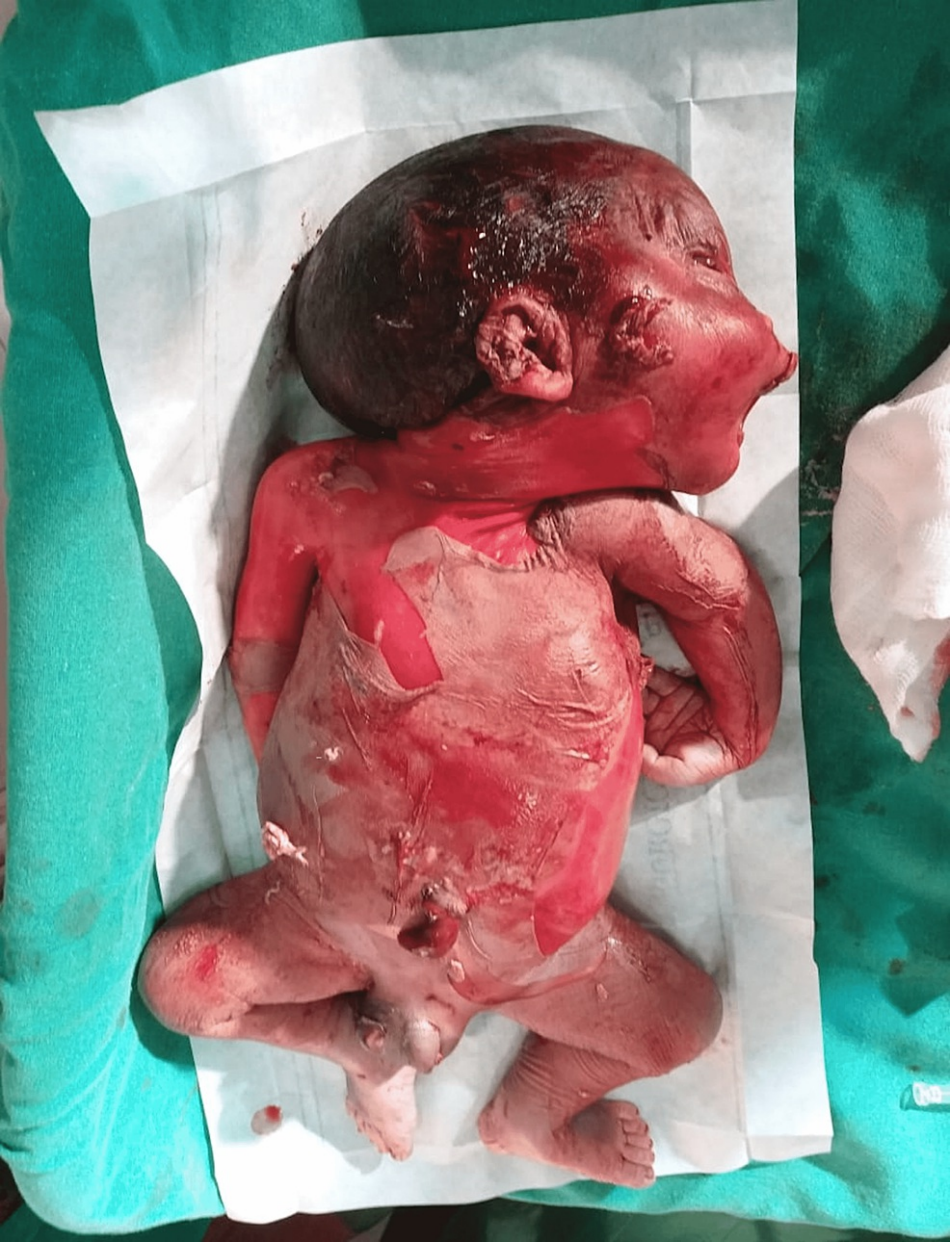
Dead macerated male baby

She was admitted and started on tablet labetalol 100 mg twice daily. Induction of labor was done with tablet mifepristone 200 mg per orally followed 36 hours later by tablet misoprostol 50 μg per vaginally every four hours for two doses. She expelled a dead macerated male fetus weighing 1825 g vaginally (Figure 6).

**Figure 7:**
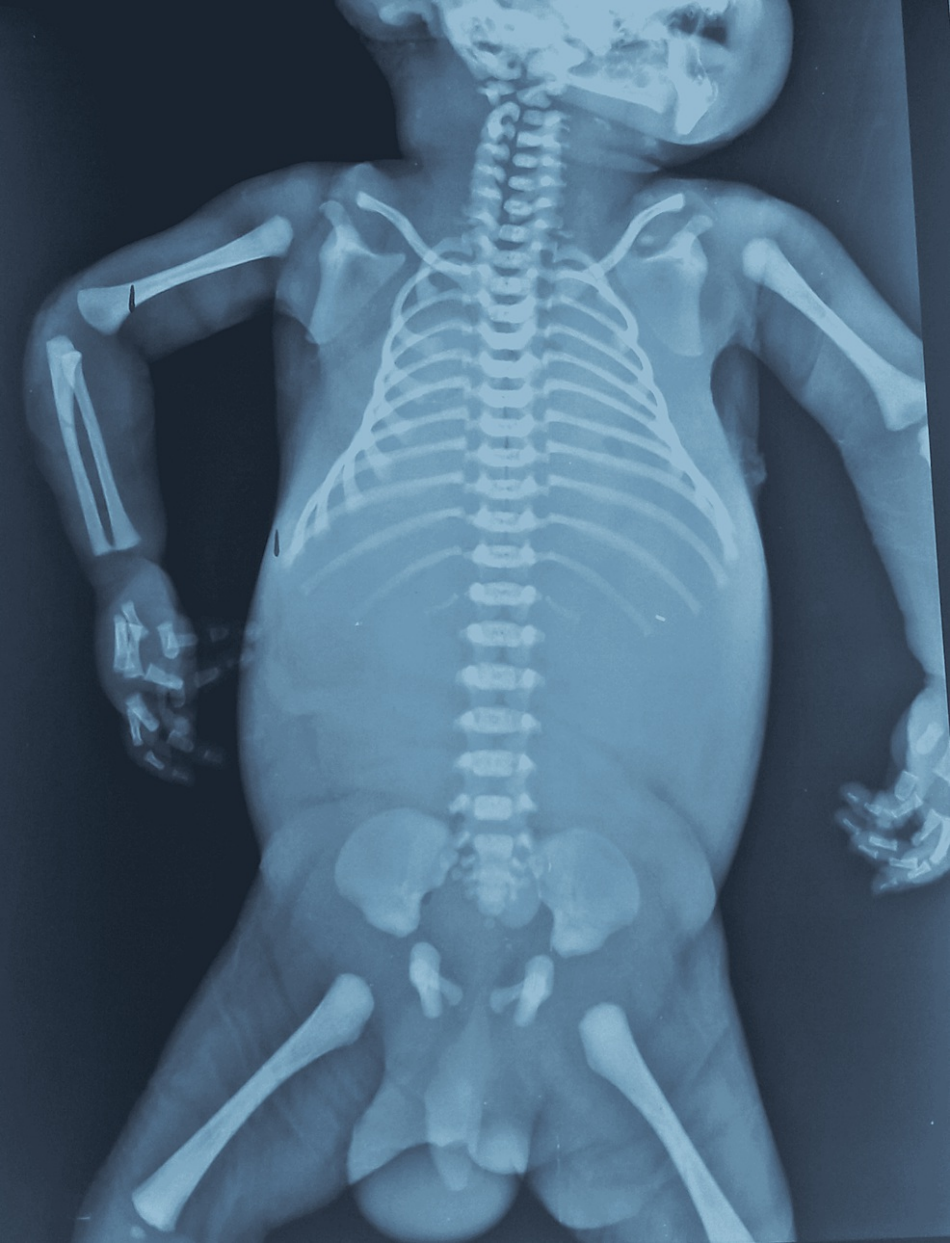
Normal infantogram of the baby

The infantogram was normal as shown in Figure 7.

**Figure 8:**
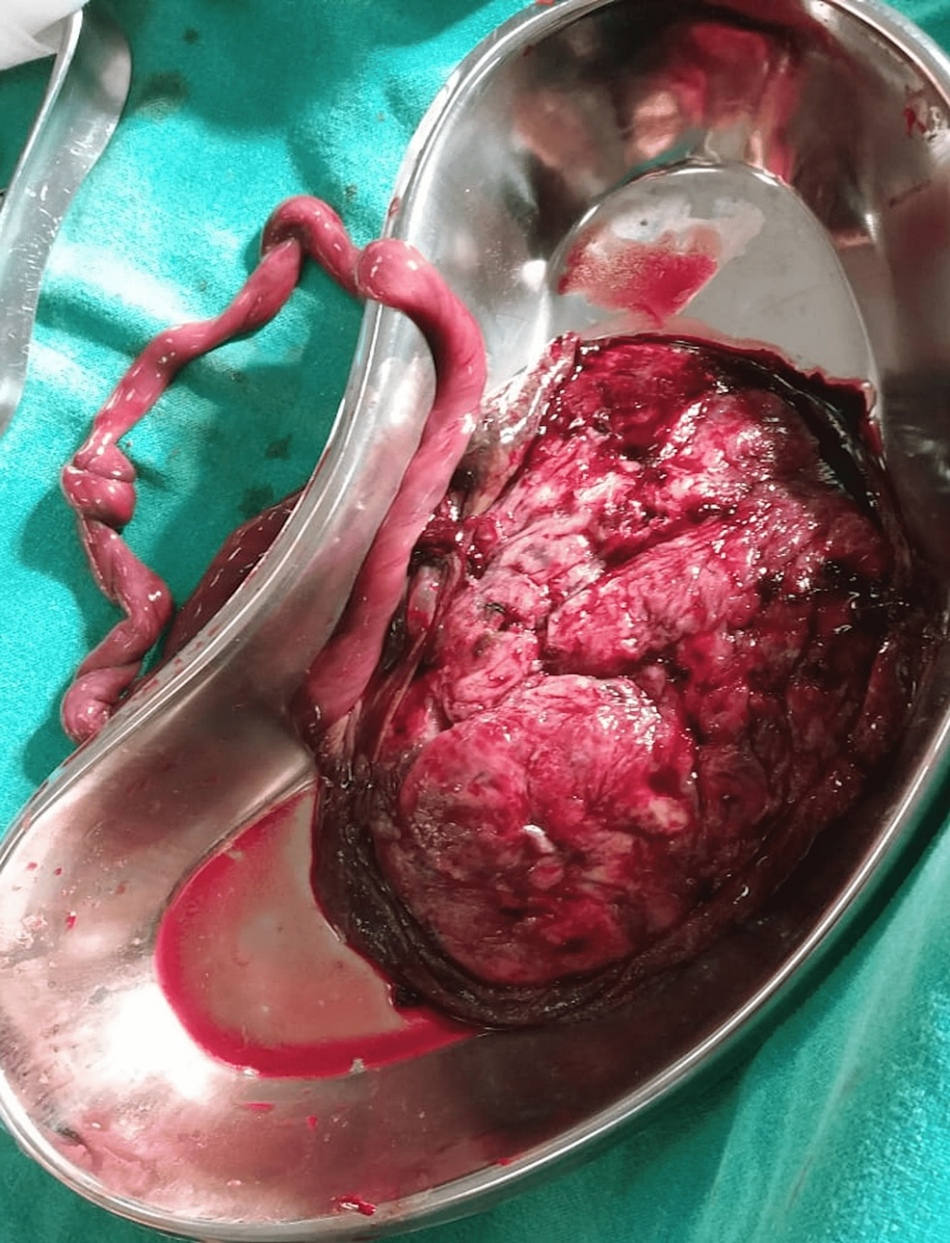
Placenta with a true knot in the cord

A true umbilical cord knot was found as shown in Figure 8.

## Discussion

The umbilical cord is the source of fetal blood supply; therefore, any cord abnormality can have a significant impact on the fetal outcome. It is predicted that TKUC usually forms between nine and 12 weeks of gestation when an adequate amount of liquor and significant fetal movements allow the fetus to slip through a cord loop [4]. But, most commonly, complications occur in later gestation when blood supply to the fetus is compromised due to cord tightening associated with fetal movements and decreasing amount of liquor with increasing gestation.

There are various factors that can predispose to TKUC, such as polyhydramnios, increased cord length, monoamniotic twins, male baby, grand multiparity, small fetus, and amniocentesis [5]. Notably, in our case, two women were multigravidas, and all three women delivered male babies. However, cord length was in the normal range for all three. It is postulated that male fetuses have more activity leading to the formation of TKUC. TKUC can lead to various adverse outcomes in pregnancy and labor like SGA fetus, low Apgar score at birth, fetal hypoxia, and fetal demise [3]. TKUC increases the risk of fetal demise by as much as four times [6-8]. In our case series, we had one case of non-reassuring fetal status at term, one case of FGR with AEDF in the UA, and one case of intrauterine fetal demise attributable to TKUC.

With the development of advanced techniques such as three-dimensional/four-dimensional color Doppler ultrasounds, TKUC can be diagnosed antenatally in the form of a four-leaf-clover, a “hanging-noose sign” (transverse section of umbilical cord surrounded by a cord loop), or by an unusual multicolor pattern in the cord [1]. The prenatal detection rate of TKUC is only 12% [3]. It mostly remains undetected unless visualized incidentally [4]. Though there is no specific management of these cases [9], a good clinical outcome can be achieved if TKUC is diagnosed antenatally and monitored closely until fetal maturity is attained [10].

## Conclusions

Although TKUC is not a rare condition and can have potentially serious outcomes, the importance of its antenatal diagnosis has still not been clearly determined. Hence, TKUC should be suspected in patients with risk factors, and emphasis should be placed on its antenatal diagnosis on ultrasonography to avoid untoward fetal outcomes and obstetric disasters in otherwise low-risk pregnancies. Though there is no specific management of these cases, a good clinical outcome can be achieved if TKUC is diagnosed antenatally and the pregnancy is monitored closely until the delivery of the fetus.
